# Adjunctive rifampin therapy for native valve *Staphylococcus aureus* endocarditis in a neonate: A case report and literature review

**DOI:** 10.1002/ccr3.4902

**Published:** 2021-10-04

**Authors:** Mohammed A. Almatrafi, Nouf Alsahaf, Elaf J. Alsharif, Jamal A. Sayed, Abdulwahab M. A. Telmesani, Dhuha Alidrisi, Alkhayat Mohammed, Rafat Mosalli, Ahmed Albaihani

**Affiliations:** ^1^ Department of Pediatrics Umm Al Qura University Makkah Saudi Arabia; ^2^ Department of Pediatrics Security Forces Hospital Makkah Saudi Arabia; ^3^ Medical College of Umm Al Qura University Makkah Saudi Arabia; ^4^ Department of Pediatric Cardiology, Maternity and Children Hospital Makkah Saudi Arabia

**Keywords:** infective endocarditis, neonatal, rifampin

## Abstract

Infective endocarditis in neonates can be fatal. Adjunctive rifampin therapy might be effective as salvage therapy in critically ill patients with *Staphylococcus aureus* native valve endocarditis (NVE). We present a case of a full‐term neonate with NVE who had a favorable clinical outcome after adding rifampin to standard therapy.

## Graphical abstract

Adding rifampin in a critically ill neonate with native valve *Staphylococcus aureus* endocarditis might be effective and lifesaving. Further studies are needed to determine rifampin's effect on neonatal *Staphylococcus aureus* native valve endocarditis.

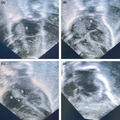

## 1 BACKGROUND

Infective endocarditis (IE) is an uncommon infection of the endothelial surface lining the heart and its associated structures.^1^, ^2^ These infections are life‐threatening, with reported mortality rates as high as 10%.^1^, ^2^ The pathogenesis of IE involves fibrin‐platelet deposits on damaged or denuded endothelium caused by abnormal cardiac structures or turbulence leading to the formation of nonbacterial thrombotic endocarditis (NBTE).^3^ NBTE acts as an excellent nidus of infection for circulating bacteria and fungi. *Staphylococcus aureus* and fungi are the most common isolated microorganisms in neonatal IE.^4^

Previous research has established that IE is less common in children than adults in recent years. An annual incidence rate for IE estimated between 0.05 and 0.12 cases per 1000 pediatric admissions. The exact incidence of IE in neonates is unknown; however, a recent multicenter study concluded that around 7% of pediatric IE was diagnosed during the neonatal period. Prematurity, structural cardiac defects, cardiac surgeries, and central venous catheters are well‐established risk factors for neonatal IE.^5^, ^6^, ^7^

Once blood cultures have been obtained, appropriate empirical antimicrobial therapy should be initiated. The optimal course of therapy and the duration are determined by the clinical response, the identified pathogen, and antimicrobial susceptibility.^5^, ^8^ Congestive heart failure, valvular dysfunction, embolic phenomena, and persistent bacteremia despite appropriate antibiotic therapy may indicate surgical intervention.^5^, ^8^

We report a case of a full‐term male who presented with fulminant sepsis, multiorgan failure, and native valve endocarditis secondary to methicillin‐susceptible *Staphylococcus aureus* (MSSA). He had a favorable clinical outcome after combination therapy with intravenous oxacillin, gentamicin, and rifampin.

## 2 CASE PRESENTATION

A late preterm baby boy of 36 weeks +5 days born to a 26‐year‐old G2P1 via spontaneous vaginal delivery was admitted to the neonatal intensive care unit (NICU) for persistent hypoglycemia secondary to transient hyperinsulinemia. The patient required a high dextrose infusion through an umbilical venous catheter (UVC) up to day 10 of life. On day 11, the patient developed respiratory distress and lethargy. On examination, the patient appeared very ill‐looking, with respiratory distress and generalized anasarca. The abdomen was tense and distended, with an erythematous indurated skin rash around the umbilicus stump at the UVC insertion site. A sepsis work‐up including blood culture and urine culture was collected, and the patient started on meropenem (20 mg/kg/dose every 8 h) plus vancomycin (15 mg/kg/dose every 8 h) empirically for neonatal sepsis and omphalitis.

Initial laboratory results showed marked leucocytosis (39 × 10e3/μL), with a predominant neutrophil count (80%), anemia (8.4 g/dL), and profound thrombocytopenia (16 × 10e3/μL). The hepatic panel revealed normal liver enzymes (AST 32.7 unit/L, ALT 24.7 unit/L), hypoalbuminemia (2.3 g/L), and direct hyperbilirubinemia (total bilirubin 24.7 mg/dL, direct 22.2 mg/dL). The coagulation profile was notable for prolonged INR (2.4), PT (31.8 s), and aPTT (45 s). Initial renal function tests were within the normal limits for the patient age group. Blood culture grew methicillin‐susceptible staph aureus (MSSA) and Escherichia coli (*E. coli*). The following day, a new grade III systolic murmur appeared in his cardiac examination, and his oxygen requirement had significantly increased to the point where he required mechanical ventilation and was eventually placed on high‐frequency oscillatory ventilation. He received numerous blood transfusions, platelet transfusions, fresh frozen plasma, and an albumen infusion for disseminated intravascular coagulopathy and hypoalbuminemia.

**FIGURE 1:**
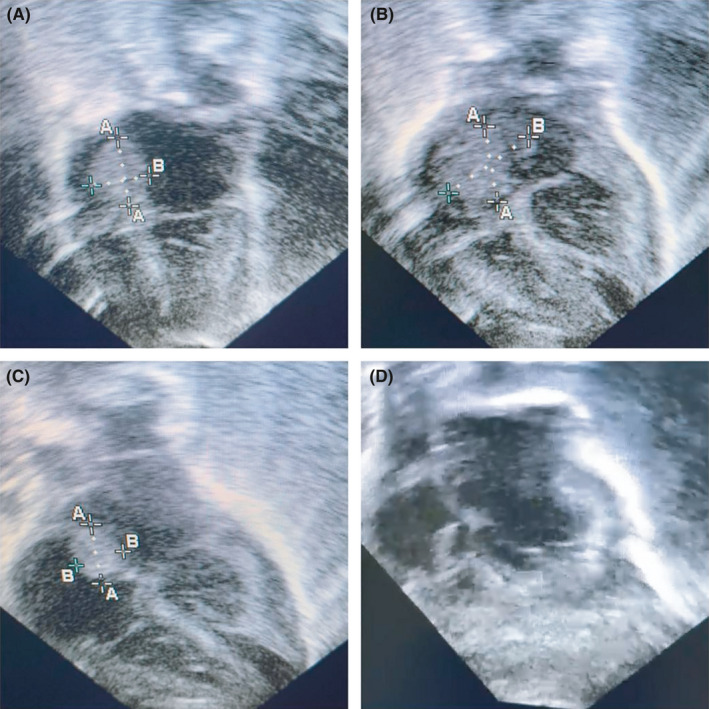
(A) Transthoracic ECHO revealed a large mass measuring 0.8 * 1 cm attached to the right interatrial septum and protruding into the left atrium. (B) A follow‐up ECHO after five days showed the progression of the vegetation size to 1.3 * 1.6 cm. (C) Significant regression in the size of the vegetation to 1.2 * 1 cm after adding rifampin. (D) Within 3 weeks of receiving combined antimicrobial therapy with rifampin, the vegetations were completely resolved without valvular dysfunction

**FIGURE 2:**
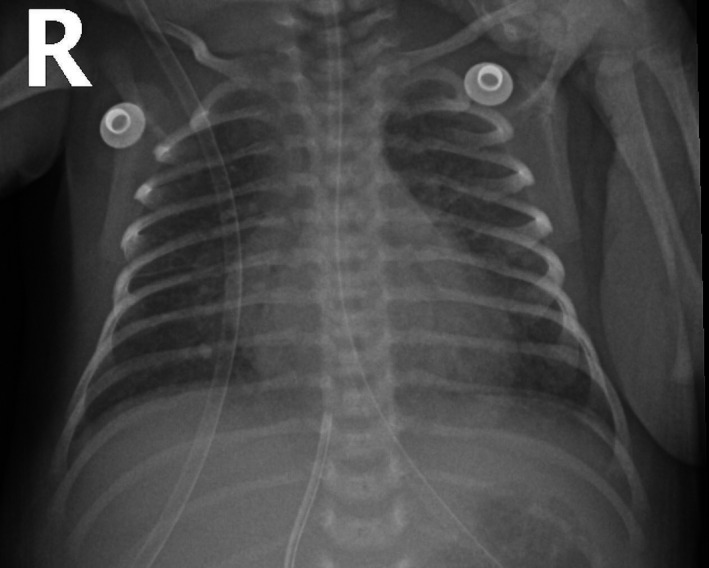
X‐ray of the chest showing bilateral reticulonodular opacities in both lung fields

An echocardiography showed a mass (0.8 * 1 cm) attached to the septum protruding to the left atrium with vegetation (Figure 1), and his chest X‐ray showed bilateral reticular densities of both lung fields, raising the possibility of showering septic emboli (Figure 2). Although the patient's *E. coli* bacteremia cleared very quickly, his blood culture for MSSA remained positive. The antibiotic therapy was changed to oxacillin (50 mg/kg/dose every 6 h) and gentamicin (5 mg/kg/dose every 24 h) for more targeted therapy for MSSA endocarditis, and cefotaxime (50 mg/kg/dose every 8 h) for *E. coli* bacteremia and presumed meningitis as LP was not obtained, given the patient's clinical instability.

Despite the patient's appropriate therapy for MSSA endocarditis, he continued to have positive blood cultures for MSSA and enlarging vegetations (1.3 * 1.6 cm) (Figure 1). The patient was not a candidate for surgical intervention with his compromised respiratory status and multiple organ failure. Rifampin (10 mg/kg/day every 24 h) was added to his regimen for its excellent tissue penetration and as a last resort therapy for his uncontrolled infection. Shortly after starting rifampin, his MSSA bacteremia took a longer time to grow in his blood culture. Eventually, the patient's clinical status improved with antibiotic therapy of oxacillin, gentamicin, and rifampin, which was evident in the cleared bacteremia and gradual reduction in the size of the vegetations (Figure 1). On day 28 of life, his ECHO was negative for vegetations (Figure 1). The patient received 2 weeks of gentamicin and rifampin from the first negative culture. Rifampin was discontinued early, and oxacillin was switched to cefazoline (130 mg/kg/day every 8 h) because of liver toxicity (ALT 990, AST 1300). The patient completed 6 weeks of antibiotic therapy from the first negative blood culture and was discharged in a stable condition.

## 3 DISCUSSION

Infective endocarditis (IE) is associated with significant morbidity and mortality.^5^ It is commonly caused by *Staphylococcus aureus*, coagulase‐negative Staphylococci, and Candida species in neonates.^5^ Clinical manifestations of infectious endocarditis vary and may include a new or changing heart murmur, respiratory distress, clinical sepsis, and an embolic phenomenon.^6^

Clinical and laboratory signs of infection in the presence of UVC raised the suspicion of IE in this case. IE was diagnosed based on the presence of two major Duke criteria: multiple positive bacterial cultures for the IE typical microorganism, and echocardiographic evidence of a mass and vegetative lesions.

The American Heart Association (AHA) current standard of care for methicillin‐susceptible *Staphylococcus aureus* (MSSA) endocarditis in a native valve or other native cardiac tissue is four to 6 weeks of intravenous antistaphylococcal penicillin (nafcillin/oxacillin), with the optional use of gentamycin for first 3–5 days. Cefazolin is an alternative in patients with penicillin‐allergic reactions. Vancomycin is used for patients who are unable to tolerate β‐lactam antibiotic drugs. Patients with methicillin‐resistant *Staphylococcus aureus* (MRSA) endocarditis are treated with vancomycin for a minimum of 6 weeks. Rifampin is recommended as an adjunctive treatment in staphylococcal endocarditis involving a prosthetic valve.^5^

This case report discusses rather unusual management of infective endocarditis in a neonate as there are no guidelines that particularly address the use of rifampin in MSSA endocarditis involving a native valve. Rifampin is a highly lipid‐soluble bactericidal agent that can achieve high intracellular concentration with excellent biofilms and tissue penetration.^9^ Therefore, current guidelines for prosthetic valve endocarditis and hardware‐associated osteomyelitis secondary to staphylococcal infections support the adjunctive use of rifampin.^5^, ^10^ Rifampin is associated with various adverse effects, including gastrointestinal symptoms, hepatotoxicity, nephrotoxicity, thrombocytopenia, hemolysis, anaphylaxis, and increased risk of drug interactions.

There has been conflicting data regarding the adjunctive use of rifampin in staphylococcal bacteremia in both adults and children. In the multicenter, randomized controlled trial (ARREST), adjunctive rifampin had no overall benefit compared with standard treatment for *Staphylococcus aureus* bacteremia in adults.^12^ Additionally, a recent meta‐analysis concluded that adding rifampin to the treatment of *Staphylococcus aureus* bacteremia in adults with deep infections did not result in a reduction in the rate of bacteriologic failure, relapse, or death.^13^

Data on the adjunctive use of rifampin in neonatal endocarditis secondary to *Staphylococcus aureus* are scarce. Tan et al. reported successful use of intravenous rifampin with vancomycin for ten neonates with persistent staphylococcal bacteremia.^14^ Within 24 h of initiating rifampin, eight of ten neonates in the study had sterile blood culture, one neonate within 48 h, and one neonate within 5 days.^14^ Among the study's notable observations were the absence of significant adverse effects of rifampin in this age group.^14^ O'Callaghan C and McDougall reported four cases of neonatal IE caused by *Staphylococcus aureus*. Two patients had complete clinical recovery when rifampin was added to their regimen, with the caveat that one patient had rifampin for a brief period.^15^

In our case, chest X‐ray findings were consistent with septic emboli and persistent bacteremia for more than 7 days and enlarging vegetation despite appropriate antimicrobial therapy are all indications for surgical intervention. However, due to his unstable general condition, he was not a candidate for surgery. Rifampin was added to his regimen as salvage therapy to control his infection. Soon after initiating rifampin, our patient's overall clinical status, as well as radiological and microbiological data, improved significantly.

## 4 CONCLUSION

This report, along with previous reports, suggests that rifampin may be clinically effective as adjunctive therapy in critically ill neonates with native valve *Staphylococcus aureus* endocarditis. Elevated hepatic transaminases could be interpreted as an adverse effect of rifampin in neonates, as demonstrated in this case. Although we believe our patient's increase in liver enzymes was multifactorial, it is critical to weigh the risks and benefits before initiating rifampin in neonates.

## CONFLICTS OF INTEREST

The authors declare no conflict of interests.

## AUTHOR CONTRIBUTIONS

MA led and designed the study, wrote, contributed to the literature search, reviewed, and edited the manuscript. NA and EA participated in the manuscript writing, literature search, and data collection. JS, AT, DA, and AM contributed to the interpretation of data and manuscript revisions. RM and AA critically revised the manuscript. All authors approved the final manuscript.

## ETHICAL APPROVAL

I testify that my article was submitted to the Clinical Case Reports Journal.

Title: Adjunctive Rifampin Therapy for Native Valve *Staphylococcus aureus* Endocarditis in a Neonate: A Case Report and Literature Review.

1. This material has not been published in whole or in part elsewhere.
2. The manuscript is not currently being considered for publication in another journal.
3. I have been personally and actively involved in substantive work leading to the revised manuscript and will hold themselves jointly and individually responsible for its content.

## CONSENT

Informed consent was obtained from the parents of this patient.

## Data Availability

The data supporting the findings of this study are available within this article.
